# Case Report: Prognostic evaluation of immunotherapy in two patients with SMARCA4-UT using TCR as a new marker

**DOI:** 10.3389/fimmu.2025.1618118

**Published:** 2025-09-29

**Authors:** Xiayu Wang, Yiwei Zhou, Qijiao Li, Jun Wang, Yu Qian, Bing Yang

**Affiliations:** ^1^ Dept. of Thoracic Oncology, Hubei Cancer Hospital, Wuhan, China; ^2^ Kindstar Global Precision Medicine Institute, Wuhan, China

**Keywords:** SMARCA4, TCR, lung cancer, non-small cell lung carcinoma, check-point inhibitors, TILs

## Abstract

Thoracic SMARCA4-deficient undifferentiated tumour(SMARCA4-UT) is a newly classified subgroup of non-small cell lung cancer (NSCLC) that is rare and associated with a poor prognosis. There is a paucity of robust research regarding its treatment options and prognostic assessment. Generally, the first-line treatments for NSCLC patients with SMARCA4 are similar to those for soft tissue sarcoma (STS). STS is partially insensitive to chemotherapy and lacks specific targeted therapeutic interventions. Consequently, the immunotherapy is increasingly applied as the first-line treatment. This paper described the treatment process of two SMARCA4 patients receiving a combination of chemotherapy and immunotherapy, employing T-cell Receptor-sequencing technology(TCR). From the perspective of the immune microenvironment, we propose a novel potential marker—TCR—that may serve as an indicator for prognostic evaluation of immunotherapy in SMARCA4 patients, thereby providing a theoretical foundation from the perspectives of tissue and peripheral immunity.

## 1 Introduction

SMARCA4-UT is a rare malignant tumor associated with a poor prognosis. Recent clinical research identified SMARCA4 as a tumor suppressor gene, which typically exhibits a small cell/rhabdoid morphology, often accompanied by BRG1 loss. In lung cancer, mutations in the SMARCA4 gene account for 12% of non-oncogene-addicted lung adenocarcinomas, while 5% of gene mutations occur concurrently in oncogenic lung adenocarcinomas (1). Given that only a limited number of SMARCA4-UT patients present with *EGFR* mutations (2), the efficacy of targeted therapy remains uncertain. In clinical practice, platinum-containing chemotherapy is frequently combined with immune checkpoint inhibitors (ICIs). The conventional biomarkers for ICIs are relatively well-established, including Programmed Death-Ligand 1(PD-L1), tumor mutational burden (TMB), and high microsatellite instability (MSI-H). Among these, only MSI-H has been approved by the US Food and Drug Administration for screening patients for pan-tumor ICI treatment. However, the incidence of MSI-H in non-small cell lung cancer (NSCLC) patients is exceedingly low (3), and the predictive value of PD-L1 expression and high TMB for neoadjuvant immunotherapy in NSCLC is highly debated (4, 5). However, those biomarkers are not fit for SMARCA4-UT patients in ICI clinical treatments. Since ICI combined chemotherapy significantly improved the median progression-free survival (PFS) compared to typical chemotherapy as first-line treatment (6), and the above biomarkers are not approved to fit for SMARCA4-UT patients in ICI clinical treatments, searching for the proper biomarkers of SMARCA4-UT in ICI therapy would benefit the patients.

This article presents two cases of SMARCA4-UT, utilizing TCR-seq technology to elucidate the biological basis for the markedly different clinical outcomes of the two patients undergoing ICI treatment, with a focus on the immune microenvironment. Furthermore, it proposes a TCR diversity index as a potential prognostic marker for SMARCA4-UT patients’ treatment outcomes.

## 2 Case description

Patient 1, a 57-year-old male, presented to the hospital on November 1, 2022, with “intermittent hemoptysis for more than 2 months.” He had no history of smoking or other medical conditions, and his ECOG performance status was 2. Initial Contrast-Enhanced Thoracoabdominal Computed Tomography scans revealed tumor lesions in the right subhilar region and the right lower lung, along with multiple mediastinal lymph nodes. A diagnosis of lung adenocarcinoma was established through a CT-guided puncture biopsy of the right lung mass. Immunohistochemical (IHC) results indicated: CD56(-), CK7(-), KI67(+; 70%), with potential for transfer, as well as NapsinA (-), P40(+), TTF-1(-), SYN (weak +), CgA (-), CK (+), EMA (+), PAS (-). Genetic testing revealed an *EGFR* E19 deletion mutation and a PD-L1 tumor proportion score of 2.4%. Based on the 8th edition of the WHO diagnostic criteria and the results of brain magnetic resonance imaging (MRI), the patient was diagnosed with stage cT3N2Mx right lung adenocarcinoma. Patient 1 started osimertinib-targeted therapy in December 2022, and two months later, he experienced a worsening of chest tightness and shortness of breath, accompanied by swallowing obstruction, anorexia, and fatigue. Additionally, new subcutaneous nodule metastases developed on the left anterior chest wall, along with metastases in the bilateral supraclavicular, axillary, hilar, and mediastinal lymph nodes. Considering the above-mentioned newly developed clinical symptoms and the findings of imaging examinations, disease progression is suspected, which may be attributed to primary resistance to third-generation EGFR-TKIs, a second left anterior chest wall puncture biopsy of the surface mass was performed in February 2023, followed by genetic testing to identify potential drug-resistant mutations. Pathological analysis confirmed the malignancy of the tumor, and based on the immunophenotype, it was classified as a SMARCA4-deficient undifferentiated tumor. Notably, the biopsy history indicated right lung adenocarcinoma, suggesting poorly differentiated cancer with dedifferentiation (SMARCA4-deficient) and the presence of an *EGFR* E19del mutation. The results of the second immunohistochemistry (IHC) analysis revealed the following tumor cell characteristics: TTF-1 (8G7G3/1)(+), CK5/6(-), CK7(-), P40(-), Ki67 (Clone: SP6)(Li: 90%), CD56(-), CgA(-), SYN (weak +), Brg-1(-), INI-1(+), PCK(+), Claudin-4(-), SMARCA2(-), SOX2(-).

Subsequently, Patient 1 received one cycle of paclitaxel (albumin-bound) plus carboplatin chemotherapy on February 12, 2023., supplemented with the PD-1 inhibitor camrelizumab, resulting in a slight relief of chest tightness. To further enhance efficacy, afatinib (40 mg, once daily) was administered in combination after chemotherapy. A review of the chest CT revealed that some lesions had shrunk, while others had enlarged; tumor markers decreased, and the right supraclavicular lymph node metastases showed slight enlargement. However, the symptoms of chest tightness were alleviated. According to the RESIST efficacy evaluation, the outcome was classified as stable disease (SD), and the treatment was tolerated well. Due to elevated transaminases and the potential adverse reactions associated with targeted therapies, afatinib was discontinued, and another cycle of paclitaxel (albumin-bound) plus carboplatin chemotherapy was administered alongside camrelizumab on March 7, 2023. After 10 days, Patient 1 experienced severe chest tightness, with worsening anterior symptoms. Notably, the left anterior chest wall metastasis nodule and right clavicle lymph nodes had enlarged compared to previous assessments. Additionally, the patient exhibited sinus tachycardia, with blood oxygen saturation ranging from 80% to 90% despite nasal oxygen inhalation. Additionally, elevated D-dimer was observed. Given the rapid progression of the tumor and the deterioration of the patient’s condition, a switch to salvage chemotherapy was initiated on March 31, 2023, consisting of docetaxel (120 mg, Day 1) and cisplatin (40 mg, Days 1-3). Throughout the treatment, Patient 1’s symptoms, including palpitations, chest tightness, breathlessness, and nasal congestion, failed to improve, and symptoms of superior vena cava compression worsened. The heart rate was approximately 120 bpm, with blood pressure readings of 75/55 mmHg in both upper limbs and 140/90 mmHg in both lower limbs. Patient 1 survived for six months following the initial diagnosis.

Patient 2 is a 51-year-old male with a history of heavy smoking who presented to the hospital with an “intermittent dry cough for one month.” The results of contrast-enhanced thoracoabdominal computed tomography (CT) and brain magnetic resonance imaging (MRI) showed that a mass in the left upper lobe of the lung measuring 3.9 cm by 3.6 cm, along with metastasis to the right adrenal gland (10.5 mm by 6.3 mm), compression of the right lobe of the liver, right kidney, and inferior vena cava, as well as right cerebellar metastasis. A CT-guided percutaneous lung biopsy was performed on February 24, 2023. The pathological diagnosis of the biopsy specimen from the left upper lung mass indicated a malignant tumor. The immune phenotype was consistent with SMARCA4 deletion undifferentiated carcinoma. Immunohistochemical results showed: P40 (-), CK5/6 (+), TTF-1 (8G7G3/1) (-), Napsin A (-), CK7 (+), Brg-1 (-), PCK (+), POU2F3 (6D1) (-), CD56 (-), CgA (-), SYN (-), INSM1 (+), Claudin-4 (+), SOX2 (+), SMARCA2 (+), INI-1 (+), and Ki67 (Clone: SP6) (Li: 70%); special stain: PAS (+). The final diagnosis was SMARCA4-deficient left lung cancer, classified as cT2aNxM1c, with metastasis to the right adrenal gland and brain, designated as stage IVB. The PD-L1 test returned negative for PD-L1 with a TPS <1%. No genetic testing was performed.

**Figure 1:**
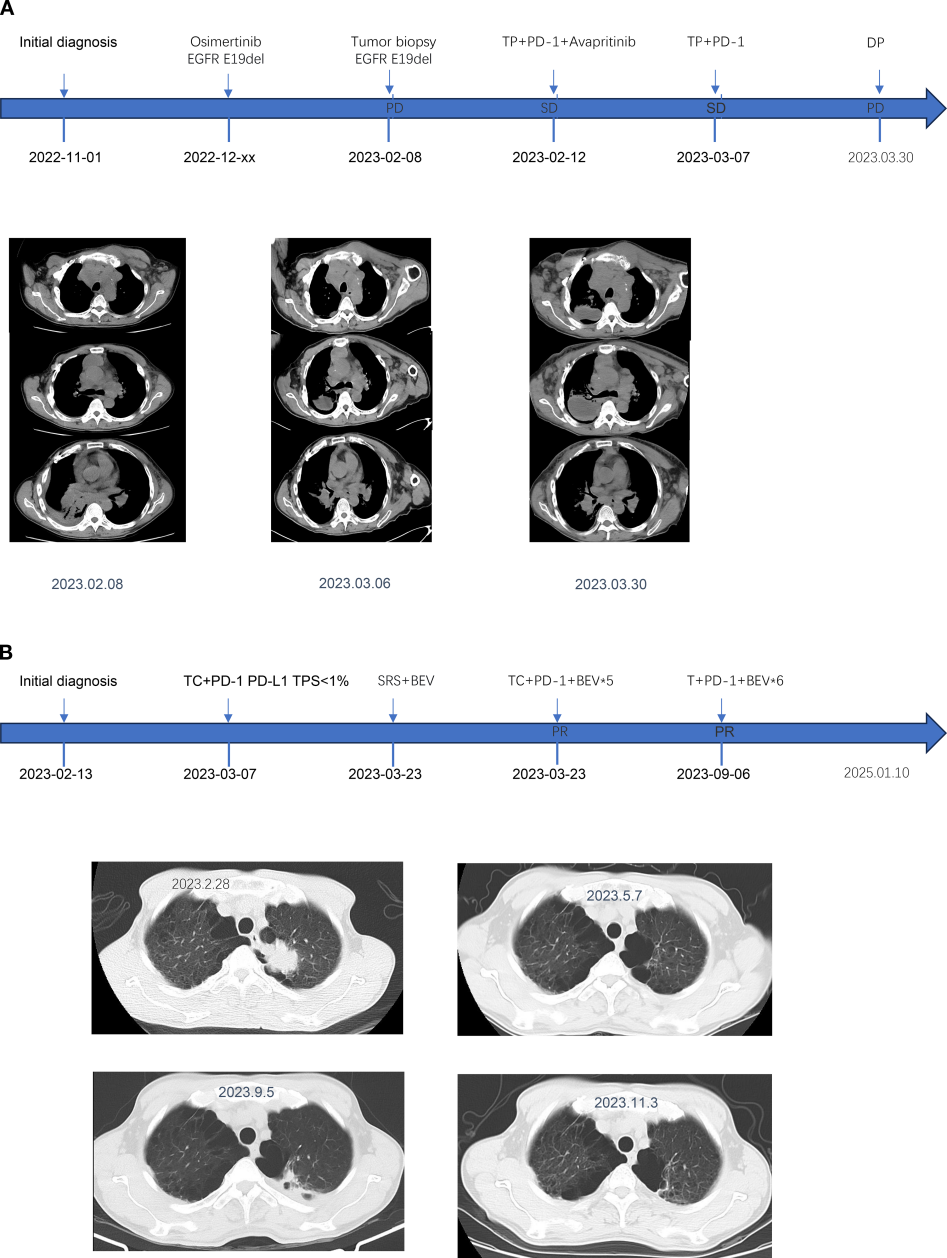
The medication regimen, diagnostic conclusions, and clinical status assessments for Patient 1 **(A)** and Patient 2 **(B)** are presented at various time points.

Two weeks after Patient 2 completed the first cycle of Taxotere+ Carboplatin(T+C)+Sintilimab treatment, he developed symptoms indicative of brain metastases, including dizziness, nausea, and vomiting. Following additional treatment with bevacizumab, the patient underwent brain IMRT radiotherapy. The neurological symptoms were pronounced. After improvement, he continued with five cycles of TC+Sintilimab+bevacizumab treatment. Upon review, the lesions exhibited shrinkage, after which he was transitioned to maintenance therapy with single-agent chemotherapy paclitaxel liposome, along with Sintilimab and bevacizumab. The patient’s survival period ultimately reached nearly 21 months. To date, there have been no immune-related adverse reactions reported during the treatment. The treatment timeline and therapeutic response are shown in **Figure 1**, and the baseline clinical characteristics, treatment regimens, and key immunohistochemical results of the two patients are summarized in **Table 1**.

**Table 1:** Baseline clinical characteristics, treatment regimens, and key immunohistochemical findings of two patients with SMARCA4-UT.

Clinical and Pathological Indicators	Case1/patient 1	Case2/patient 2
Age,y/Sex	57/M	51/M
Smoking status	Former smoker	Former smoker
TNM	cT3N2M1,IVA	cT2aNxM1c, IVB
Treatment	Chemotherapy+PD-1+Osimertinib	Chemotherapy+PD-1+Bev
Overall Survival outcome	6 mo	21 mo
TTF-1, CK5/6,CK7, P40 Ki67 CD56 CgA SYN Brg-1 INI-1 PCK Claudin-4 SMARCA2 SOX2 Driver Gene Mutation (EGFR/ALK/ROS1)	TTF-1(8G7G3/1)(+), CK5/6(-), CK7(-), P40(-), Ki67(ClONE: SP6)(Li:90%), CD56(-), CgA(-), SYN(+), Brg-1(-), INI-1(+), PCK(+), Claudin-4(-), SMARCA2(-), SOX2(-)	TTF-1(8G7G3/1)(-), CK5/6(+),CK7(+), P40(-), Ki67(ClONE: SP6)(Li:70%), CD56(-), CgA(-), SYN(-), Brg-1(-), INI-1(+), PCK(+), Claudin-4(+), SMARCA2(+), SOX2(灶+),
*EGFR/ALK/ROS1*	*EGFR* 19del	NA

The treatment strategies for the two patients included chemotherapy, targeted therapy, and immunotherapy; however, their final survival outcomes differed significantly. To enhance the clinical understanding of the efficacy and prognosis of SMARCA-UT patients, we conducted a comprehensive evaluation of the tumor immune microenvironment. We utilized bulk TCR-seq technology to analyze the infiltration of immune T lymphocytes in tumor tissues and peripheral blood, and to assess related TCR clonal types. Previous studies have employed TCR-seq immune repertoire analysis to evaluate TCR diversity and types, providing insights into the immune status of patients across various diseases. This technique has been applied in multiple cancer types, including bladder cancer and non-small cell lung cancer, and has shown prognostic prediction capabilities, particularly in the context of immunotherapy strategies, thereby aiding in the establishment of patient prognosis assessment models.

**Figure 2:**
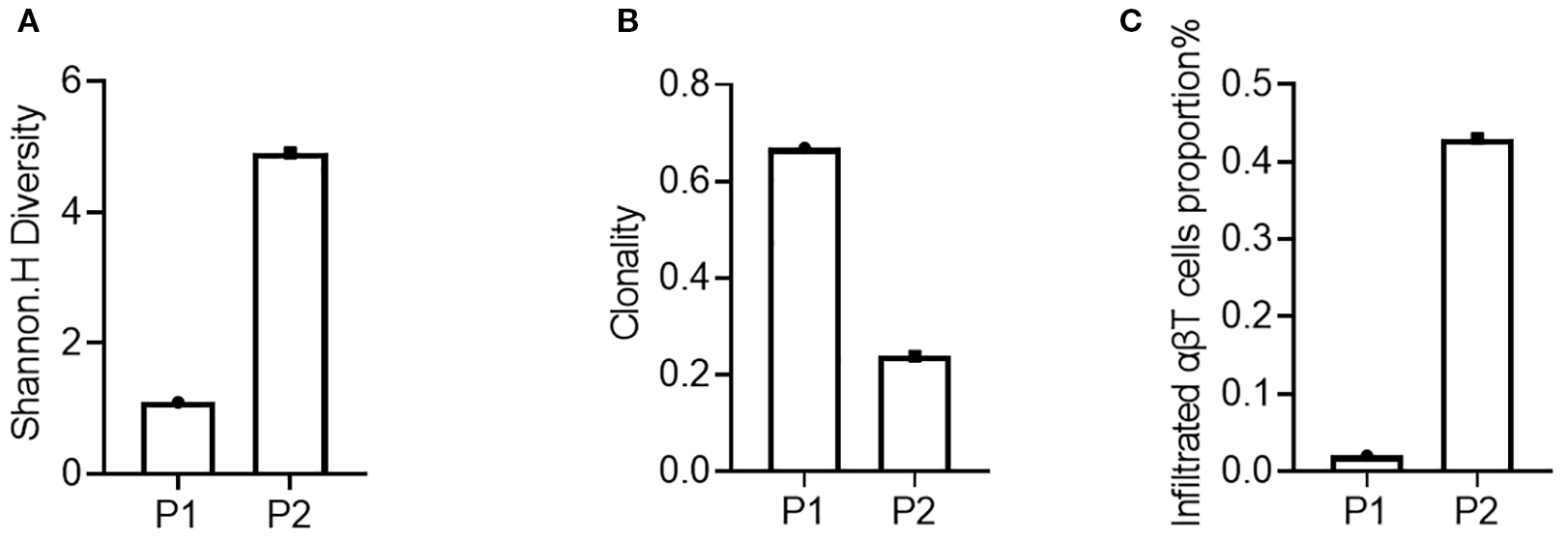
Assessment of the immune microenvironment of the patient’s tumor tissue before treatment. **(A)** Diversity of the P1’s and P2’s tumor tissues; **(B)** Clonality of the P1’s and P2’s tumor tissues; **(C)** Proportion of infiltrating lymphocytes in the P1’s and P2’s tumor tissues.

In this study, TCR-seq results indicated the proportion of T lymphocyte infiltration in the tumor tissue, allowing for an evaluation of immune infiltration for both patients. Specifically, the proportion of T lymphocyte infiltration in the tumor tissue of patient 2 (P2) was 0.43% (**Figure 2**), while that of patient 1 (P1) was only 0.02%, suggesting that patient 2’s tumor immune microenvironment exhibited a stronger immunosuppressive state. Furthermore, TCR-seq results also revealed the diversity of T lymphocytes in the tumor tissue (**Figure 2**), demonstrating that patient 1 had significantly lower TCR diversity compared to patient 2 (**Figure 2**).

**Figure 3:**
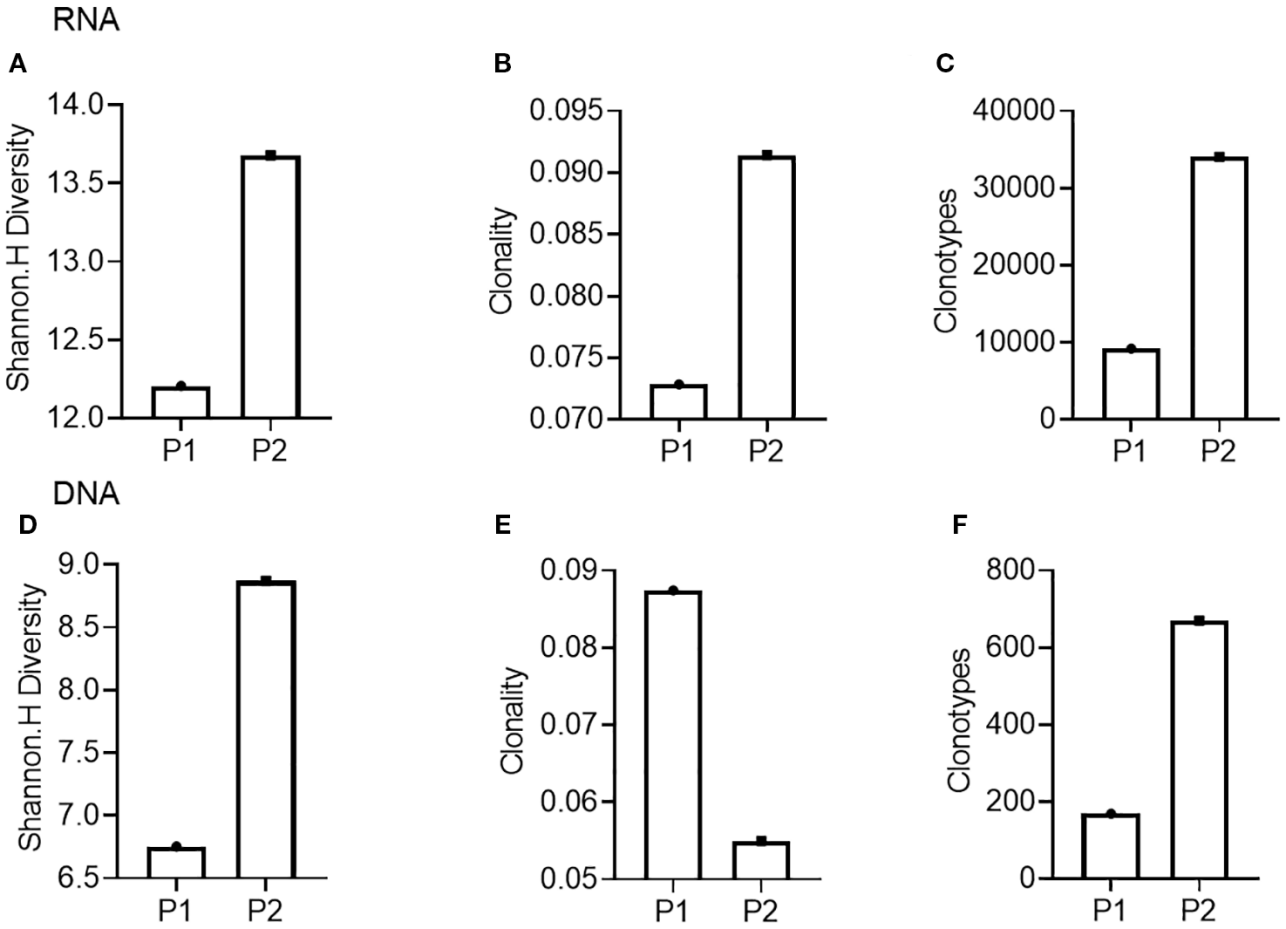
Comparison of TCR diversity indices in peripheral blood of patients before treatment. **(A-C)**, TCR diversity **(A)**, clonality **(B)**, and clone type **(C)** of Patient 1 (P1) and Patient 2 (P2) compared using RNA as template; **(D, E)**, TCR diversity **(D)**, clonality **(E)**, and clone type **(F)** of Patient 1 (P1) and Patient 2 (P2) compared using DNA as template.

**Figure 4:**
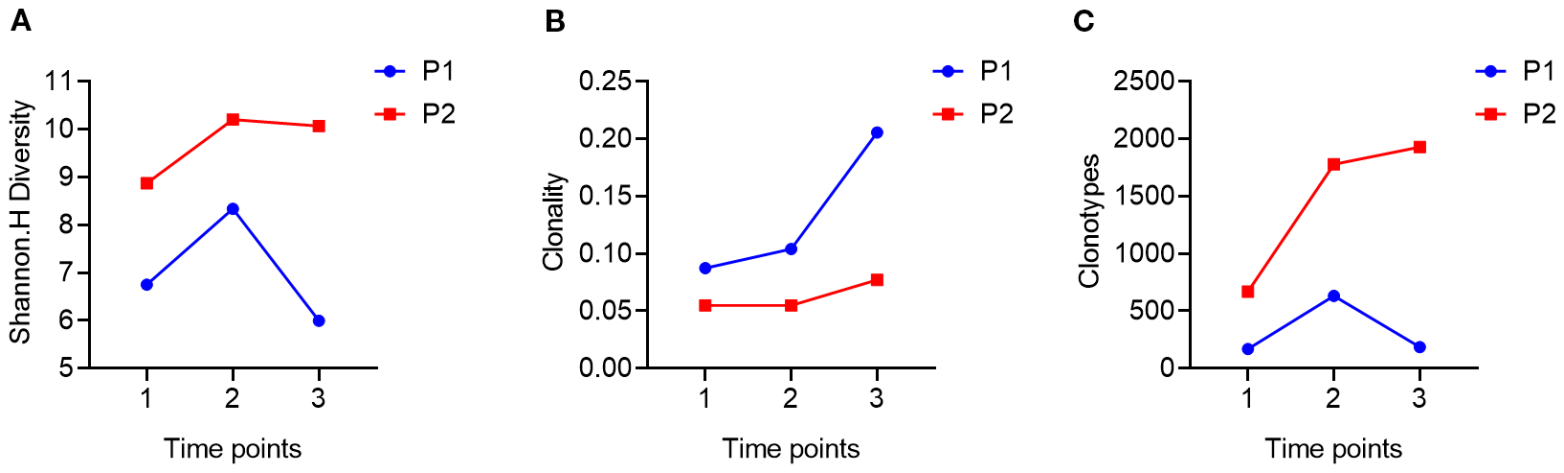
Trends in patients’ TCR diversity indices during treatment. **(A)**, using DNA as a template to compare the changing trends of TCR diversity in patient 1 (P1) and patient 2 (P2) at three time points during treatment (1.2.3); **(B)**, using DNA as a template, comparing the changes in TCR diversity in patient 1 (Trends in the clonality of TCR in P1) and patient 2 (P2) at three time points (1.2.3) during treatment; **(C)**, using DNA as a template, comparing the changes in TCR diversity in patient 1 (Trends in the number of clonal types also called clonotype of TCR in P1) and patient 2 (P2) at three time points (1.2.3) during treatment.

**Figure 5:**
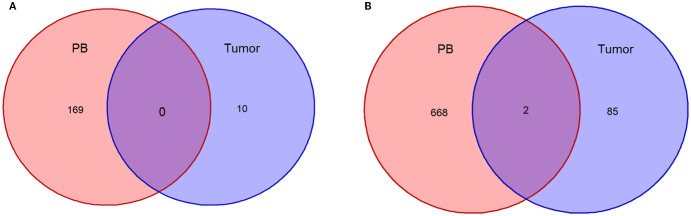
Overlap of TCR clonotypes in patients’ PB and tumor tissues before therapy. **(A)**, Plotting of T-lymphocyte clonal overlap status of peripheral blood and tumor tissue before treatment in Patient 1; **(B)**, Plotting of T-lymphocyte clonal overlap status of peripheral blood and tumor tissue before treatment in Patient 2.

Next, we conducted TCR-seq detection on the patient’s peripheral blood T lymphocytes and analyzed the TCR diversity index. The results suggested that patient 1, who has a poor survival prognosis in this report, exhibits relatively low baseline (pre-treatment) of TCR diversity, high clonality, and a low number of TCR clones, which contrasts sharply with patient 2 (**Figure 3**). Throughout the treatment process, patient 1 experienced a sharpened increase in diversity, followed by a rapid decrease. Conversely, patient 2, who presented better treatment effect had a higher baseline TCR diversity. Following treatment, the clonality increased slowly, then resulting in a more even distribution of clones and an increase in the total number of clones. During the treatment process, patient 2’s diversity initially increased and then stabilized (**Figure 4**). Finally, we compared the types of peripheral blood lymphocytes with those of tissue lymphocytes. According to previous studies, the overlap index can provide insights into the body’s immune function and may serve as a prognostic factor for immunotherapy. The findings of this study reveal that the overlap types of TCR clones between patient 1’s peripheral blood and tumor tissue was 0, while 2 of the 668 clones in patient 2’s peripheral blood overlapped with TCR clones in the tumor tissue (**Figure 5**). These results suggested that the prognosis for patient 2 may be more favorable than that of patient 1. This conclusion is consistent with current clinical information, indicating that the TCR index has the potential to predict clinical prognosis.

## 3 Discussion

In 2015, Le Loarer et al. (7) identified a group of undifferentiated rare thoracic malignant tumors characterized by SMARCA4 mutations and BRG1 deletions, which exhibited transcriptomic similarities to ovarian small cell carcinoma and high-risk tumors. Calcemic small cell carcinoma shares these characteristics. These tumors are distinguished by increased instability, frequent TP53 mutations, a higher tumor mutation burden, and the absence of germline SMARCA4 alterations, leading to the designation SMARCA4-DTS. In 2021, SMARCA4-DTS was recognized as a subgroup of NSCLC in the fifth edition of the WHO classification of thoracic tumors. The designation was changed from SMARCA4-DTS to SMARCA4-UT, and the disease was categorized into two subtypes: SMARCA4-deficient undifferentiated thoracic tumors (SMARCA4-UT) and SMARCA4-deficient non-small cell lung cancer (SMARCA4-dNSCLC) (8, 9).

Patients with SMARCA4-UT primarily seek medical attention due to mass infiltration and compression of adjacent organs, including the mediastinum, pleura, and lungs. This can lead to complications such as superior vena cava syndrome, atelectasis, spinal cord compression, and esophageal invasion, resulting in dyspnea (10). At the time of treatment, 83% of patients present with localized symptoms. The disease progresses rapidly, particularly in late and advanced stages. The median overall survival (OS) for SMARCA4-dNSCLC is 7.8 months (11), while for SMARCA4-UT, it is 5.6 months. The 2-year survival rate is 12.5%, and the median progression-free survival (PFS) time is only 30 days (12). According to Sauter et al., SMARCA4-UT has a worse prognosis than SMARCA4-undifferentiated thoracic tumors (11). In lung cancer, SMARCA4 gene mutations account for 12% of non-oncogenic addictive lung adenocarcinomas, with 5% of these mutations also present in oncogenic lung adenocarcinomas (1). Alterations in SMARCA4 define a subset of NSCLC, with approximately 10% of NSCLC cases exhibiting SMARCA4 loss. The loss of SMARCA4 is associated with the development of advanced dedifferentiated tumors and an increased incidence of tumor metastasis (7).The SMARCA4 gene encodes the BRG1 protein, and its loss is associated with a poor prognosis. Studies have demonstrated that in patients with surgically resectable NSCLC, the loss of BRG1 protein and low levels of SMARCA4 expression predict a worse prognosis, irrespective of tumor stage (13). In brief, class I mutations of the SMARCA4 gene refer to complete loss-of-function mutations that result in complete inactivation or total absence of the BRG1 protein(truncating mutations, fusions and homozygous deletion), while Class II mutations refer to those that only cause partial loss of BRG1 protein function(missense mutations) (14) with patients harboring class I mutations exhibiting poorer survival outcomes compared to those with class II mutations. However, it is noteworthy that patients with class I mutations tend to respond more favorably to ICIs (15). In recent years, SMARCA4-UT has emerged as a distinct subgroup of NSCLC, garnering increasing attention in both diagnostic and treatment approaches. The primary distinction between SMARCA4-UT and SMARCA4-dNSCLC lies in their histological differentiation and immunohistochemical characteristics. SMARCA4-UT is classified as an undifferentiated tumor, whereas SMARCA4-dNSCLC represents a subtype of NSCLC. Notably, SMARCA4-UT is associated with greater invasiveness and a poorer prognosis, in contrast to SMARCA4-dNSCLC, which demonstrates a clearer differentiation and a treatment response more akin to typical NSCLC. The fundamental molecular features of SMARCA4-UT include mutations in the SMARCA4 gene and deletions of BRG1, typically accompanied by a TTF1(−) phenotype. Due to the challenge of distinguishing SMARCA4-UT from other tumors based solely on clinical manifestations, its diagnosis necessitates a comprehensive approach that integrates clinical findings with laboratory testing. This includes a thorough analysis of examination results, histopathology, immunohistochemistry, and genetic mutations.

Histologically, SMARCA4-UT is characterized as a poorly differentiated tumor exhibiting rhabdoid or epithelioid features. In terms of pathological diagnosis, the current consensus holds that a diagnosis of SMARCA4-UT can be made when BRG1 immunohistochemical staining is negative, irrespective of the presence of a SMARCA4 gene mutation. Additionally, it is suggested that the TMB may be elevated in these cases (16). Furthermore, there is a hypothesis that SMARCA4-deficient NSCLC may acquire a secondary mutation, leading to the loss of BRM protein and resulting in the transformation into the more poorly differentiated SMARCA4-UT. Consequently, patients with SMARCA4-UT may also exhibit concurrent loss of BRM protein (17).Currently, there is no unified standard for treatment strategies for SMARCA4-UT. Common treatment options include surgical intervention, combination chemotherapy, radiotherapy, targeted therapy, immunotherapy, and epigenetic therapy (18). Studies suggest that the first-line chemotherapy regimen for NSCLC with SMARCA4-UT is analogous to that employed for soft tissue sarcoma (STS) (19, 19). In STS, patients receiving ICI immunotherapy exhibited an increased density of cytotoxic tumor-infiltrating T cells, a higher percentage of activated T cells, and tumor-associated macrophages expressing PD-L1. Furthermore, STS characterized by tertiary lymphoid structures (TLS) demonstrates a high response rate to ICI therapy, irrespective of their histological subtype (20). Immunotherapy is now a first-line treatment in the overall management of lung cancer, but the inclusion of immunotherapy and whether it is based on PD-L1 expression in the subgroup of SMARCA4-UT remains inconclusive. In 2022, a study conducted at the University Hospital of Strasbourg in France examined the immune desert characteristics of the tumor microenvironment (TME) in SMARCA4-UT patients by analyzing the infiltration levels of immune cells. The findings indicated that tumor lymphoid follicles may serve as potential markers for predicting responses to ICI therapy in these patients. The study revealed that patients lacking TLS positivity in the cohort exhibited poor responses to ICI and shorter OS (21) (4). This research underscores the importance of identifying effective markers as potential bioindicators of ICI response for SMARCA4-UT patients, thereby complementing the roles of PD-L1 expression and TMB in NSCLC.

In this article, the researchers reported on two patients with markedly different clinical outcomes. Pathological tissue sections and imaging analyses indicated that both patients had SMARCA4-deficient undifferentiated lung malignant tumors. Based on established evidence (22) demonstrating superior efficacy of immunotherapy combined with chemotherapy versus chemotherapy alone for second-line treatment in patients with EGFR-TKI-resistant non-small cell lung cancer (NSCLC), chemotherapy-immunotherapy combination was administered as second-line therapy, with planned discontinuation of immunotherapy upon occurrence of immune-related adverse events (irAEs).Based on the smoking history and advanced age of this male patient with lung squamous cell carcinoma (Patient 2), genetic testing was not routinely recommended per the Chinese Medical Association Lung Cancer Clinical Diagnosis and Treatment Guidelines (23, 24). Both patients received similar clinical treatment strategies, which included a combination of chemotherapy, immunotherapy, and targeted therapy. However, Patient 1 experienced a short survival period, poor prognosis, and rapid disease progression, while Patient 2 achieved an overall survival of 21 months. Previous studies suggest that the *EGFR* 19 del mutation may serve as a poor prognostic factor for SMARCA4-mutated NSCLC (14), which aligns with results of this study. Additionally, this article presents further data on the immune microenvironment, aiming to provide insights into the clinical management of patients from alternative perspectives and to explore additional markers that could serve as prognostic indicators, ultimately aiding in patient prognosis assessment. Researches have demonstrated that a high density of tumor-infiltrating T lymphocytes (TILs) is a favorable prognostic biomarker and is associated with responses to immunotherapy (25).

A substantial number of studies have categorized solid tumors into three phenotypes based on the degree of immune infiltration: immune infiltrate (infiltrate), immune rejection (exclude), and immune desert (desert). Immune-infiltrated tumors exhibit a higher density of T cells, particularly CD8^+^ T cells, within their structure. In contrast, immune rejection tumors display a greater accumulation of T cells at the peripheral edge, where these cells express elevated levels of the cell surface glycoprotein serine protease (FAP). This expression regulates the formation of a dense extracellular matrix surrounding the tumor, thereby hindering the infiltration of immune cells into the tumor’s interior. The immune desert phenotype is characterized by a near-total absence of T lymphocyte infiltration (26–29).

Consequently, this study introduces TCR-seq technology to assess the level of tissue immune infiltration in patients with SMARCA4 mutations. Currently, TCR-seq technology has not yet reached a mature stage of application in first-line clinical settings; however, previous studies have demonstrated its prognostic value in patients with bladder cancer (30), non-small cell lung cancer (31), melanoma (32), and other diseases. Not surprised, those studies don’t indicate the same trends in TCR diversity or clonality in clinical outcomes prognosis. But the findings regarding TCR diversity index values before and after patient treatment suggest significant clinical evaluation potential in establishing a patient prognosis assessment model (33–35). In our study, we firstly connected the association of SMARCA4 patients’ prognosis and the TCR diversity in both PBMC and tumor infiltrated T cells via TCR-seq technology (8, 33), exploring the immune microenvironment of the timepoint before ICB therapy of patients’ and the dynamic changes in PBMC. Although the technology is not the first time applied in solid tumor prognosis prediction, our work still offered the novel indications in SMARCA4 NSCLC therapy.

Our report not only evaluates the immune infiltration cells numbers and types that presented in patients’ tumor tissues but also monitors the peripheral blood immune diversity index of these patients. The peripheral blood TCR diversity index is recognized as an indicator of patient prognosis in clinical studies, with multiple investigations demonstrating that higher peripheral blood TCR diversity correlates with improved prognosis and extended survival. This report indicated that Patient 2 exhibited higher peripheral blood TCR diversity prior to treatment, which was associated with a better treatment prognosis (36). In contrast, Patient 1’s peripheral blood TCR diversity before treatment was significantly lower than that of Patient 2, correlating with a poorer treatment outcome. This suggests that peripheral blood TCR diversity may serve as a potential prognostic indicator for the efficacy of immunotherapy in SMARCA4 patients. However, it’s easy to realize that the TCR clonality showed slight differences in RNA level compared with DNA level in two patients, which probably due to the technology we applied, multiplex PCR amplification can amplify these highly transcribed clones and thereby inflate their apparent frequency, causing the clonality bias.

Additionally, this study dynamically monitored the peripheral blood TCR diversity of patients throughout the treatment process. The results revealed that Patient 1’s TCR diversity initially increased significantly but then decreased sharply over three treatment cycles. Conversely, Patient 2’s TCR diversity exhibited a pattern of initial increase followed by stabilization during the same period, with both patients maintaining stable clonality after the initial increase. This phenomenon may suggest the immune homeostasis contributed to better clinical outcome of ICI in SMARCA4 NSCLC. Numerous previous studies have arrived at varying conclusions regarding the relationship between changes in TCR diversity and disease prognosis. Some research indicates that elevated TCR diversity prior to treatment is associated with a favorable prognosis, particularly in patients exhibiting a high TMB, as greater TCR diversity may signify enhanced antigen recognition capabilities (37). Conversely, other studies have demonstrated that an increase in TCR clonality following treatment—characterized by the expansion of a specific T cell clone—correlates more closely with a positive prognosis. It is suggested that, while initial TCR diversity is important, the expansion of specific T cell clones during treatment may play a crucial role in mediating anti-tumor immunity. This difference in results highlights the necessity of considering not only the impact of TCR diversity prior to treatment on prognosis but also the clinical significance of dynamic changes in TCR during treatment with respect to disease control and treatment response. Furthermore, multiple studies have indicated that significant variations in immune status can occur across different treatment courses and stages. Consequently, analyses conducted at a single time point may yield inconsistent conclusions, complicating the accurate correlation of laboratory data with real-world clinical outcomes. To more comprehensively evaluate the dynamic changes in TCR profiles, an effective approach is to compare the overlap index of T cell clonal types infiltrating the patient’s tissue with those present in the peripheral blood circulation. A high overlap index typically indicates an active tumor antigen-specific immune response, and relevant studies have demonstrated that a high TCR overlap index is strongly associated with favorable prognoses in patients undergoing treatment with immune checkpoint inhibitors, such as PD-1/PD-L1 inhibitors (38). Furthermore, the dynamic monitoring of changes in TCR clone enrichment or diversity during treatment can provide additional insights into therapeutic efficacy and the mechanisms underlying tumor immune evasion.

In this report, patient 1 exhibited an overlap index of 0, while patient 2 demonstrated the presence of two T cell clones in both peripheral blood and tumor tissue. This report presents the cases of two patients diagnosed with SMARCA4 non-small cell lung cancer. We combined gene mutations with the expression of the traditional immune marker PD-L1 to investigate the markedly different prognoses of immunotherapy in these patients. More importantly, we conducted an in-depth analysis of the TCR diversity index as a reflection of the immune microenvironment, highlighting its therapeutic prognostic potential in SMARCA4 non-small cell lung cancer. We propose the TCR diversity index as a potential marker for predicting treatment outcomes, thereby providing valuable insights for the clinical evaluation of patient management.

## Data Availability

The datasets presented in this study can be found in online repositories. The names of the repository/repositories and accession number(s) can be found in the article/supplementary material.
