# Correction: Case Report: Prognostic evaluation of immunotherapy in two patients with SMARCA4-UT using TCR as a new marker

**DOI:** 10.3389/fimmu.2025.1717357

**Published:** 2025-10-10

**Authors:** Xiayu Wang, Yiwei Zhou, Qijiao Li, Jun Wang, Yu Qian, Bing Yang

**Affiliations:** ^1^ Dept. of Thoracic Oncology, Hubei Cancer Hospital, Wuhan, China; ^2^ Kindstar Global Precision Medicine Institute, Wuhan, China

**Keywords:** SMARCA4, TCR, lung cancer, non-small cell lung carcinoma, check-point inhibitors, TILs

A correction has been made to the author list. The “equal contributions symbol” was erroneously excluded from authors “Xiayu Wang and Yiwei Zhou”. The department for affiliation 2 has also been removed.

The corrected author list and affiliation appears below:

“Xiayu Wang ^‡^¹, Yiwei Zhou ^‡^², Qijiao Li², Jun Wang¹, Yu Qian¹, Bing Yang¹*

¹Dept. of Thoracic Oncology, Hubei Cancer Hospital, Wuhan, China

²Kindstar Global Precision Medicine Institute, Wuhan, China

^‡^These authors contributed equally to this work and share first authorship”.

